# Retraction Note: Multisensory learning binds neurons into a cross-modal memory engram

**DOI:** 10.1038/s41586-026-10355-4

**Published:** 2026-03-25

**Authors:** Zeynep Okray, Pedro F. Jacob, Ciara Stern, Kieran Desmond, Nils Otto, Clifford B. Talbot, Paola Vargas-Gutierrez, Scott Waddell

**Affiliations:** 1 Centre for Neural Circuits & Behaviour, University of Oxford, Oxford, UK; 2 Present Address: Institute of Anatomy and Molecular Neurobiology, Westfälische Wilhelms-Universität Münster, Münster, Germany

Retraction to: *Nature* 10.1038/s41586-023-06013-8 Published online 26 April 2023

The authors have retracted this article. After conducting replication experiments and reanalysing the original data, the authors discovered that the voltage imaging results, as presented in Figs. 3a through 3f and Figs. 4a and b, and discussed in the “Neurons gain cross-modal activation” section, are not reproducible. Additional voltage imaging work by the authors and their collaborators, for the conditions associated with Figs. 3a through 3d, has not produced robust signals. Furthermore, errors in the pipeline originally used for data analysis could have led to the contamination of the Δ*F*/*F*_0_ data. A complete reanalysis by the authors of the voltage imaging data independently from the original pipeline did not replicate the original analysis. Although the authors have replicated the behavioural and connectomic data and consider that the general conclusions of the article are substantiated, the authors have no confidence in the voltage imaging data. The authors would like to apologize for any inconvenience caused to their scientific colleagues and the readers of the journal. The follow-up data described above will be made available to the community as soon as possible.

The authors would like to thank Waddell lab members Anna Cook, Ashwin Miriyala, and Martín Klappenbach, who were involved in the replication experiments but are not authors of the original paper, and Max Hoffmann, Stephen Thornquist, and Gaby Maimon of Rockefeller University for identifying possible errors in the voltage data processing.

All authors agree with the retraction, with the exception of Pedro F. Jacob, who could not be contacted by the corresponding author.

